# Reduction of motion artifact in pulse oximetry by smoothed pseudo Wigner-Ville distribution

**DOI:** 10.1186/1743-0003-2-3

**Published:** 2005-03-01

**Authors:** Yong-sheng Yan, Carmen CY Poon, Yuan-ting Zhang

**Affiliations:** 1 Joint Research Center for Biomedical Engineering, The Chinese University of Hong Kong, Shatin, Hong Kong

## Abstract

**Background:**

The pulse oximeter, a medical device capable of measuring blood oxygen saturation (SpO2), has been shown to be a valuable device for monitoring patients in critical conditions. In order to incorporate the technique into a wearable device which can be used in ambulatory settings, the influence of motion artifacts on the estimated SpO2 must be reduced. This study investigates the use of the smoothed psuedo Wigner-Ville distribution (SPWVD) for the reduction of motion artifacts affecting pulse oximetry.

**Methods:**

The SPWVD approach is compared with two techniques currently used in this field, i.e. the weighted moving average (WMA) and the fast Fourier transform (FFT) approaches. SpO2 and pulse rate were estimated from a photoplethysmographic (PPG) signal recorded when subject is in a resting position as well as in the act of performing four types of motions: horizontal and vertical movements of the hand, and bending and pressing motions of the finger. For each condition, 24 sets of PPG signals collected from 6 subjects, each of 30 seconds, were studied with reference to the PPG signal recorded simultaneously from the subject's other hand, which was stationary at all times.

**Results and Discussion:**

The SPWVD approach shows significant improvement (p < 0.05), as compared to traditional approaches, when subjects bend their finger or press their finger against the sensor. In addition, the SPWVD approach also reduces the mean absolute pulse rate error significantly (p < 0.05) from 16.4 bpm and 11.2 bpm for the WMA and FFT approaches, respectively, to 5.62 bpm.

**Conclusion:**

The results suggested that the SPWVD approach could potentially be used to reduce motion artifact on wearable pulse oximeters.

## Introduction

Wearable medical devices are capable of continuously monitoring an individual's vital signs in real time. These devices are particularly important to the world's increasingly aging population, whose health conditions have to be assessed regularly or monitored continuously. The devices can warn individuals of symptoms of deterioration, e.g. alerting them when their blood pressure is increasing to a level above a predetermined threshold. The devices can also automatically notify emergency services in critical situations. In order to make wearable devices practical, a series of technical problems have to be solved. For example, these devices need to be miniature in size, must possess a user-friendly interface and be efficient in power consumption. Most importantly, these devices need to have a low failure rate and must report minimal false alarms. In other words, these devices are required to provide an accurate estimate of the monitored vital sign under normal daily life situations. This leads to the important topic on the reduction of motion artifacts [1-4]. In this paper, the smoothed pseudo Wigner-Ville distribution (SPWVD) is investigated as a novel motion artifacts resistant approach for estimating one of the most important vital signs – the blood oxygen saturation level (SpO2).

The paper is organized as follows. Section 2 reviews the techniques commonly used for attenuating motion artifacts in pulse oximetry. Section 3 discusses the basic theory for SpO2 computation and the techniques used in this study for reducing motion artifacts. Section 4 compares the performance of two time-frequency techniques, i.e. the short-time Fourier transform (STFT) and the SPWVD. Section 5 presents the protocol and the results of an experiment to assess motion artifact reduction in real data. Section 6 discusses the performance of the SPWVD approach as compared to the traditional time domain and spectral methods. Lastly, the major findings of this paper are summarized in section 7.

## Background

SpO2 is commonly monitored by a pulse oximeter, which has been widely adopted as a standard measure during anesthesia, neonatal care and post-operative recovery [5,6]. Pulse oximeters currently available on the market normally perform remarkably well when the monitored subject is in the resting position. However, their reliability is significantly reduced when the subject moves, even when movements are only involuntary, such as shivering [1-4,7]. Therefore, the reduction of motion artifacts is of particular concern in the development of pulse oximeters to be applied in ambulatory, pediatric and trauma settings, as well as for implementing them into wearable devices for personal home healthcare [8].

A number of attempts have been made in the past decade to improve the accuracy of pulse oximeters when subjects move. Typical methods can be generally classified into three categories: (1) based on an independent measure of motion; (2) based on a model of the ideal signal or the noise; and (3) based on features recognized from the corrupted signal. For techniques based on an independent measure of motion, one or more transducers (e.g. piezo or optical sensors) are employed to record the user's motion. By assuming that the artifact is a linear addition to the pulsatile photoplethysmographic (PPG) signal, the original signal can be reconstructed from the corrupted signal [9-11]. This hypothesis is however often doubted when inspecting PPG signals under typical artifact-producing forces [12]. This observation drives researchers to develop more realistic models for the PPG signal or the artifact.

A recently proposed PPG artifact reduction methodology was based on the inversion of a nonlinear physical artifact model and could significantly reduce the effect of changes of probe coupling [8,12]. However, model-based techniques suffer inherently from the specificity of the model design and are unable to cope with all aspects of real-life scenarios.

On the other hand, techniques based on feature recognition are free of the generic problem of model designs. Instead, these techniques often utilize some predetermined criteria to separate regions of corrupted and uncorrupted PPG signal and estimate the desired parameters from the uncorrupted portion of it. For example, Swedlow *et al*. calculated the derivative of a signal and identified a portion of it as a motion artifact whenever the ratio of adjacent positive and negative peaks of the derivative is below a threshold [13]. J.E. Scharf *et al*. evaluated the use of spectral analysis to separate the cardiac physiologic components from the recorded PPG signal that is contaminated by motion artifact for SpO2 estimation [14-16].

The above methodologies employ techniques in the time domain or frequency domain. However, due to the nonstationary nature of PPG signals, the use of time-frequency analysis appears to be extremely attractive. Dowla *et al*. proposed using a neural network together with a wavelet transform (WT) to estimate SpO2 in the presence of a motion artifact, and found out that this technique performs better than conventional algorithm that detects peaks and troughs of the PPG signal for estimating SpO2 levels [17]. In their method, a neural network was trained to identify the motion level, which was then fed into a second neural network together with the amplitude ratios at different scales of WT of the PPG signal to estimate SpO2 levels. It has been pointed out by another researcher [16] that using WT for SpO2 computation requires careful analysis and additional testing. WT does not result in a spectrum where the amplitude of a unique cardiac frequency can be directly obtained for SpO2 estimation. On the other hand, although such a unique component is available on the spectrum obtained from fast Fourier transform (FFT), the time-frequency resolution of FFT or STFT is relatively low when compared to other time-frequency techniques such as the Wigner-Ville distribution. The goal of this study is to investigate the use of SPWVD, a high resolution time-frequency transformation where the amplitude of a unique cardiac frequency is apparent, for the estimation of SpO2 levels.

## Methods

### Basic theory

The traditional algorithms for estimating SpO2 detect peaks and troughs of the PPG signal in the time domain. Based on the Beer-Lambert law, which relates the optical path length and effective absorbance to the intensity of transmitted light, the relationship between intensity of transmitted light and SpO2 is commonly described as:

*I *(*λ*, *t*) = *I*_0 _(*λ*) exp[(-*sε*_*HbO*2 _(*λ*) + (1 - *s*)*ε*_*Hb *_(*λ*))·*c*·*d *(*t*)],     (1)

where, *ε*_*HbO*2 _and *ε*_*Hb *_are the extinction coefficients of oxygenated and de-oxygenated hemoglobin, and *s*, *c*, and *d *represent SpO2, total concentration of hemoglobin and the optical path length respectively.

By using two light sources – red and infrared lights – and calculating a normalized ratio of the AC component to the DC component for each light source, SpO2 can be computed from the ratio of ratios *R*, i.e. the normalized ratio of the red to the infrared transmitted light intensity. That is,





In practice, SpO2 can be obtained from equation (3) directly or by an empirical equation that relates SpO2 and *R*. In this study, SpO2 is estimated directly from equation (3).

### SpO2 computation by weighted moving average (WMA)

By calculating the ratio of the AC components and the ratio of the DC components of the two light sources, SpO2 can be obtained from every single pulse of a PPG signal. To stabilize the reading, the weighted moving average (WMA) is often used [18]. Typical averaging methods, e.g. the median averaging and standard arithmetic averaging, are applied to every several samples or samples in every several-second intervals. In this study, the SpO2 obtained by the WMA approach was the average of SpO2 samples in an 8-second period. Overlap processing was performed at 1-second interval. The 8-second period is selected in order to satisfy clinical requirements [15,16].

### SpO2 computation by fast Fourier transform (FFT)

Based on the hypothesis that cardiac rate can be estimated more easily by spectral analysis than time domain analysis, techniques in the frequency domain have been widely investigated as alternatives in pulse oximetry. For example, the FFT and discrete cosine transform (DCT) were proposed for estimating SpO2 [14-16]. These techniques calculate the spectrogram of the PPG signal in a fixed time period and select the strongest spectral line in the cardiac frequency band as the AC component. The cardiac frequency band is usually predetermined by certain thresholds or obtained from an independent pulse rate estimator, e.g. by applying electrocardiography in parallel. In this study, FFT is applied to every 8-second PPG signal at 1-second interval. The cardiac frequency band is predetermined as 0.8–2 Hz, i.e. corresponding to 48–120 bpm.

### SpO2 computation by SPWVD

The Wigner-Ville distribution (WVD) of a signal *x*(*t*) is given as:



where *x*(*t*) and x*(*t*) are the time series of the signal and its complex conjugate respectively.

The problem of the WVD is the so-called cross-term interference, which appears as frequencies that lie between the frequencies of any two strong components. In order to suppress cross-term interference, the smoothed pseudo WVD is often used:



The two windowing operations *h *and *g *are equivalent to smoothing the WVD in the frequency and time domain respectively. Selection of the window is a compromise between the joint time-frequency resolution and the level of cross-term interference. Common choices of window include the rectangular and Kaiser windows [19-21]. In our experiment, we chose the Hamming window as both the time and frequency smoothing windows, *g*(*t*) and *h*(*τ*).

The maximum magnitude within the cardiac frequency band of the SPWVD in each second was used for SpO2 computation. Since SPWVD represents energy distribution, the square root of the magnitude was used for calculating the ratio of ratios *R *and SpO2. Moreover, as the rate of change of SpO2 is relatively slow, SpO2 that changed by more than 2% per second was considered to be physiologically impossible, and was rejected from the calculation [22].

## Simulation: STFT spectrogram versus SPWVD

The performance of SpO2 computation by the SPWVD approach was evaluated in a simulation using PPG signals collected from a volunteer under modest random motions. For comparison, SpO2 was also estimated from the spectrogram obtained by STFT.

The noise-mixing-composition (NMC) method was applied to mimic the clinical situation and to induce a range of signal-to-noise ratios (SNR) [23]. To synthesize a noise-contaminated signal, an artifact noise that has been verified to be similar to the real noise was added to an undisturbed basis signal. The synthesized signal and SNR are formulated as:



with the basis episode *S *and artifact episode *N *and a mix parameter *ε*, which was adjusted to achieve the desired SNR.

**Figure 1:**
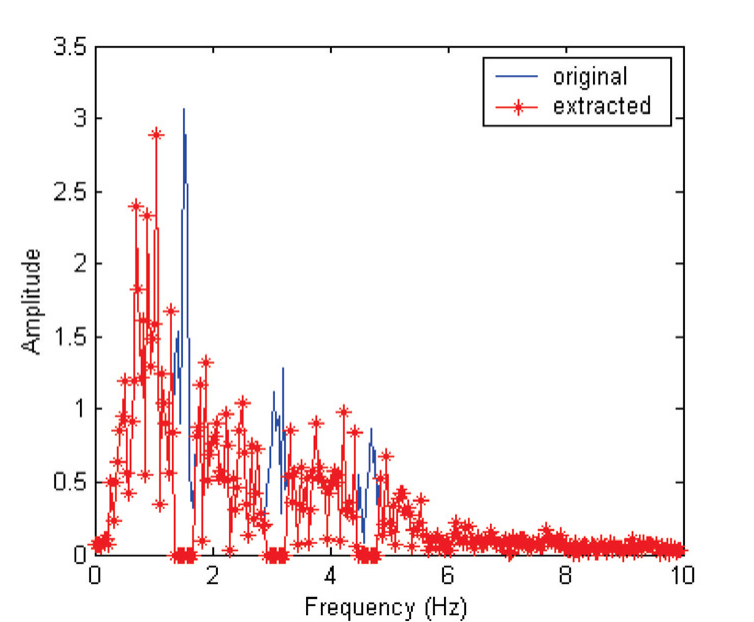
Typical spectra of contaminated signal and extracted artifact. Spectrum of the contaminated signal (solid) is obtained by FFT. Coefficients of the spectrum within 0.5 Hz of the primary cardiac frequency or its harmonics were set to zeros to result in the spectrum of pure artifact noise (starred).

512 samples of the PPG signal were recorded at 20 Hz from a volunteer. The signal was contaminated with in-band noise but with the cardiac frequency recognizable. The artifact noise was extracted by a filtering technique in the frequency domain. The spectrogram of the PPG signal was calculated using an FFT-based algorithm. Coefficients of the spectrum within 0.5 Hz of the primary cardiac frequency or its harmonics were set to zeros. The inverse FFT of the modified spectrum allowed us to obtain a pure artifact noise. Typical spectra of the contaminated signal (solid) and the resulting pure artifact noise (starred) are shown in Figure 1.

**Figure 2:**
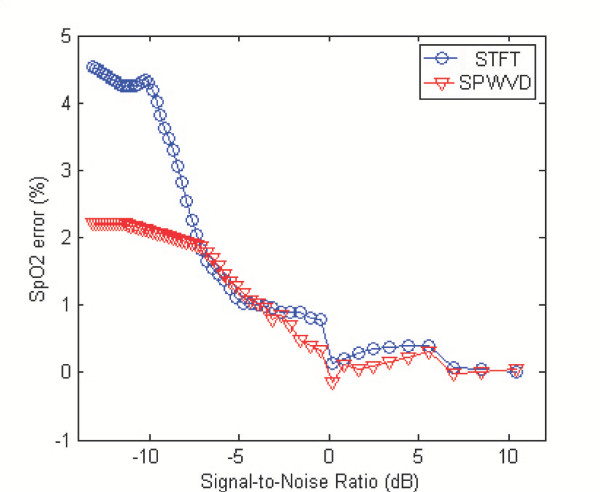
The mean SpO2 error obtained by the STFT and SPWVD approaches at different levels of SNR. The SPWVD approach outperforms the STFT-based technique for low SNR.

A set of synthesized signals with different SNR values was obtained by changing the value of the mix parameter *ε *in equation (6). The undisturbed signal was also estimated from equation (6) by setting *ε *= 0. SpO2 were estimated from the set of synthesized signals in an 8-second period at 1-second interval by using the STFT and SPWVD approaches. The mean SpO2 error during the complete 25.6 seconds is shown in Figure 2 as a function of SNR.

Figure 2 suggests that the two approaches lead to similar results for high SNR values (e.g. SNR>-5 dB). However, the SPWVD method outperforms the STFT-based technique for low SNR (e.g. SNR<-5 dB). Also, it is observed in this simulation that the errors are randomly positive or negative for high SNR values, but is mostly positive for low SNR values, i.e. the approaches consistently overestimate the SpO2 level. When the SNR value decreases, energy in the side-bands of the noise artifact that overlapped with the cardiac frequency components increases. And therefore, the resultant SpO2 level approaches a value that would have been estimated from the pure noise artifact, which differed by 7.2% from the actual SpO2 level for this specific trial.

It should be noted that the pulse rate was predetermined in the simulation, which helped both approaches to determine the cardiac frequency band more accurately. In practical situations, the electrocardiogram can be recorded simultaneously and used as a reliable pulse rate estimator. As for the computational cost, the SPWVD approach can be implemented efficiently by making use of its symmetry properties, and thus, it can reduce the computational cost to a quarter of that of the STFT technique [20].

## Experiment and Results

### Experimental protocol

The purpose of this experiment is to compare the performance of three different methods (WMA, FFT, and SPWVD) in estimating SpO2 on subjects when they are (a) in a resting position and (b) in motion.

Six healthy subjects participated in the study. Four kinds of motions have been investigated: horizontal movement (M1) and vertical movement (M2) of the hand, as well as the bending motion (M3) and pressing motion (M4) of the finger. These motions were selected because they are some of the common movements attributable to the motion artifact in pulse oximetry [7,24]. Subjects were asked to perform all four movements, 4 times each, and each time for a duration of 30 seconds. When performing each movement, subjects were asked to move their right hand, or the index finger of their right hand, for a magnitude of 2–5 cm at a frequency of 0.5–4 Hz, while keeping their left hand stationary. Signals were recorded simultaneously from the index fingers of both hands. Throughout the analysis, SpO2 or pulse rate estimated from the left hand, which was stationary at all times, was used as the reference. The reference estimates were obtained by the WMA method.

The collected signals were separated into an AC and a DC component. The AC component was filtered out by a 4th order Butterworth band-pass filter with cut-off frequencies at 0.5 Hz and 20 Hz. The ratio of the DC components was computed directly in the time domain and the same value was used for the three different approaches, i.e. the WMA, FFT and SPWVD approach. On the other hand, a different ratio of the AC components was computed using each of the three approaches.

To evaluate the performance of the different approaches, the SpO2 bias and precision, the pulse rate error, the dropout rate and the SpO2 performance index (PI) were calculated. The bias and precision are defined as the mean and standard deviation of the difference between reference and estimated SpO2 respectively. The pulse rate error is the difference between reference and estimated pulse rate. The dropout rate and SpO2 PI are evaluation parameters adopted from previous work by S.J. Barker [24]. The dropout rate is the percentage of time during which the technique fails to give a SpO2 reading, and SpO2 PI is the percentage of time during which the SpO2 level was within 7% of the reference reading.

## Results

Table 1 shows the composite values from all the experiments when subjects were in a resting position and in motion. As indicated in Table 1, all three approaches can achieve 100% SpO2 PI, 0.0% dropout rate and less than 3 bpm mean absolute pulse rate error in this experiment with a limited dataset.

**Table 1:** Performance statistics of the different approaches. The bias, precision and performance index (PI) of SpO2, as well as the mean absolute pulse rate error and dropout rate, are used to evaluate the performance of the WMA, FFT and SPWVD approaches when subjects are in a resting position and in motion.

State	Approach	SpO2 bias (%)	SpO2 precision (%)	SpO2 PI (%)	Mean absolute pulse rate error (bpm)	Dropout rate (%)
Resting	WMA	0.19	0.34	100	1.25	0.0
	FFT	0.24	0.53	100	2.51	0.0
	SPWVD	0.21	0.41	100	1.35	0.0

Motion	WMA	-1.31	3.58	81	16.4	4.6
	FFT	-1.42	3.18	83	11.2	0.0
	SPWVD	-1.07	2.42	91	5.62	0.0

However, the SPWVD approach shows significant improvement in both SpO2 and pulse rate estimation as compared to the WMA and FFT approaches when subjects were in motion. SpO2 estimated from the SPWVD, WMA and FFT approaches differed from the reference by -1.07 ± 2.42%, -1.31 ± 3.58% and -1.42 ± 3.18%, respectively. The mean absolute pulse rate error is reduced significantly (p < 0.05) from 16.4 bpm and 11.2 bpm for the WMA and FFT approaches, respectively, to 5.62 bpm for the SPWVD approach. The SpO2 PI also has the highest SpO2 PI (91%). Both the SPWVD and FFT approaches achieve 0.0% dropout rate. The WMA approach sometimes failed to give a reading during bending or pressing motions (dropout rate = 4.6%), which would lead to instrument "dropout" or "freeze" in clinical situations.

**Figure 3:**
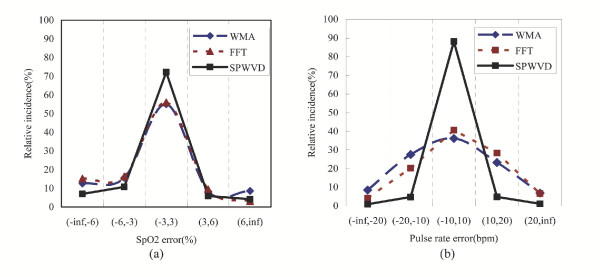
The distributions of (a) SpO2 bias and (b) pulse rate error obtained by the WMA, FFT and SPWVD approaches

Figure 3 shows the distribution of SpO2 bias and pulse rate error of the three approaches. As shown in Figure 3(a), the SpO2 errors obtained by the SPWVD approach have a higher incidence (72%) in the main error band (-3%, 3%), which is the range of bias commonly accepted by most pulse oximeter manufacturers, as compared to that obtained by the WMA (55%) and FFT (56%) approaches.

For the estimation of pulse rate, 90% of the pulse rate error falls in the error band (-10 bpm, 10 bpm) when the SPWVD approach is used (see Figure 3(b)). When compared to the WMA and FFT approaches, where only 36% and 40% of the error fall in this error band respectively, the SPWVD significantly outperforms the other two approaches.

**Figure 4:**
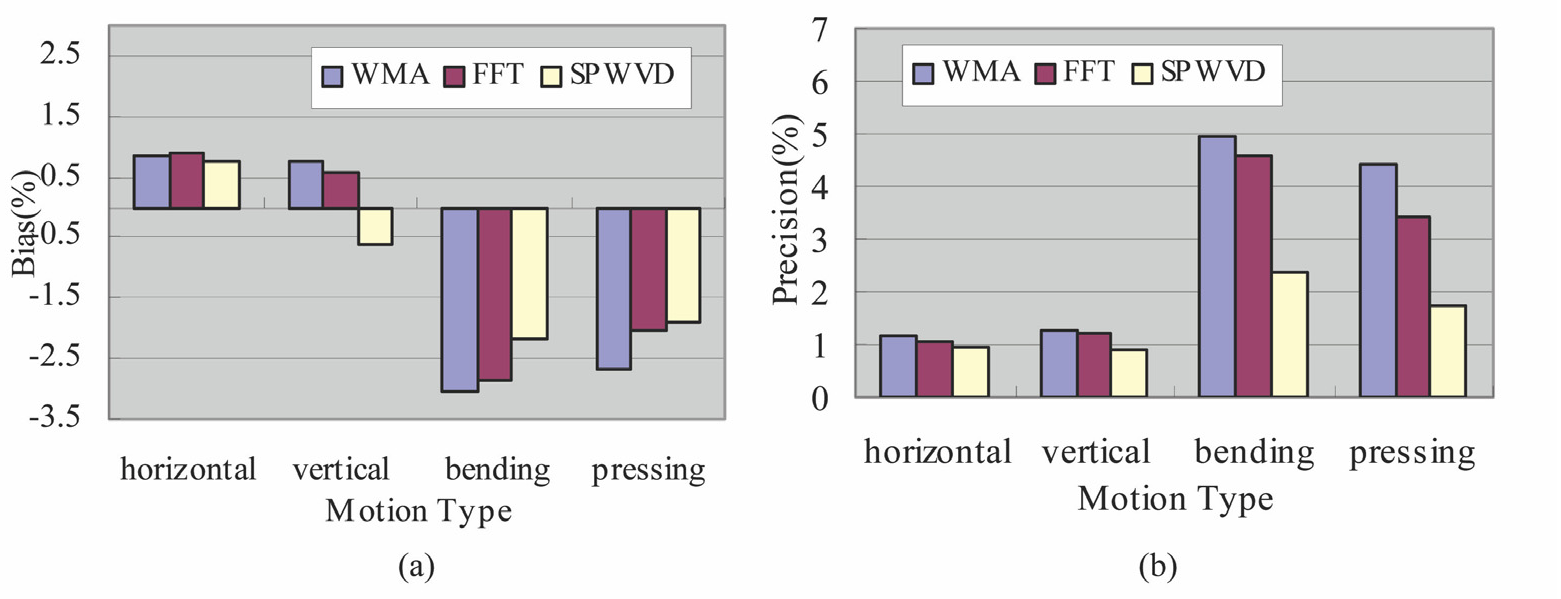
SpO2 (a) bias and (b) precision when subjects performed different types of motions: horizontal movement and vertical movement of the hand, as well as the bending motion and pressing motion of the finger.

Figure 4 shows the SpO2 output bias and precision under conditions with different kinds of motions: horizontal and vertical movements of the hand, as well as bending and pressing motions of the finger. It can be seen that the estimation of SpO2 by the SPWVD approach improved significantly (p < 0.05) as compared to the WMA and FFT approaches when subjects bend their finger or press their finger against the sensor. The three approaches show no significant differences (p > 0.05) when subjects move their hand horizontally or vertically.

**Figure 5:**
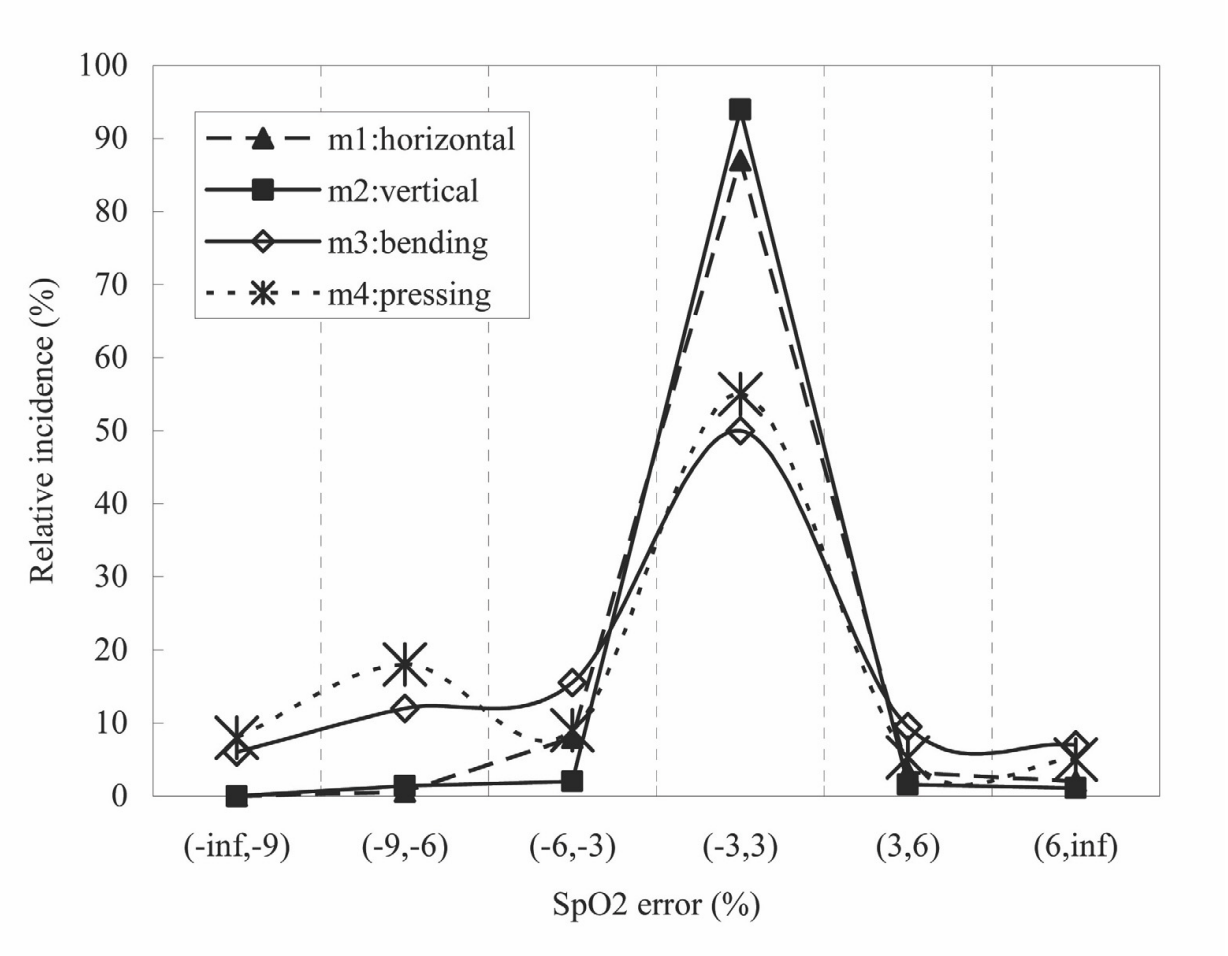
The error distributions of SpO2, obtained by the SPWVD approach, when subjects performed different types of motions: horizontal movement and vertical movement of the hand, as well as the bending motion and pressing motion of the finger.

Figure 5 gives the error distribution of SpO2, obtained by the SPWVD approach, when subjects were in different types of motions. It is found that the bending (M3) and pressing motions (M4) of the finger have a relatively broader error distribution than the horizontal and vertical movements of the hand (M1 and M2). It can also be seen that the error distribution of M2 is slightly more concentrated than that of the M1.

## Discussion

Spectral analysis is useful for separating motion artifact and cardiac physiologic spectra [14-16]. However, these techniques will not be applicable to spectra that contain frequency bands close to each other. Moreover, since both the motion and cardiac frequency are nonstationary in nature, simply using techniques in the frequency domain would not be able to separate them when one of the spectra varies within the fixed time window (i.e. an 8-second period in this study). Therefore, a time-frequency representation of the corrupted signal would be useful. The SPWVD approach is proposed for the reduction of motion artifacts because it can suppress cross-term interference while maintaining a good time-frequency concentration [19]. In addition, the approach utilizes the fact that SPWVD is an energy distribution and directly calculates the magnitude of the AC component from the spectrum. The approach solves the problem of WT, where a unique value for the cardiac frequency may not always be available [14-16]. Moreover, the approach does not require a large amount of samples for training, as the back-propagation neural network approach proposed in [17].

Standard parameters used to evaluate the performance of the techniques in pulse oximetry have been adopted in this study. S.J. Barker [24] evaluated 20 commercial pulse oximeters on 70 subjects, where data were recorded on each subject for 6 minutes during normal situation and 3 minutes during a hypoxemic episode. A motorized motion table was used to induce rubbing or tapping motions of the finger, with amplitude of ± 2 cm and frequency either fixed at 3 Hz or randomly varied between 1–4 Hz. As compared to the performance of some of the commercial products evaluated in [24], which have SpO2 bias in the range of 0.4–12%, SpO2 precision in the range of 2–6%, and SpO2 PI in the range of 27–94%, the proposed SPWVD approach reports comparable performance.

The four motions investigated in this study are some of the common movements associated with motion artifacts affecting pulse oximetry [24]. By studying the effect of each component on the estimated SpO2, one would have a clearer picture of what kind of motion induces the largest error on SpO2 estimation. In future studies, it would be interesting to develop a model that specifically deals with one type of motion. As suggested by Figure 4 and Figure 5, bending the finger (M3) or pressing the finger against the sensor (M4) induces a larger error on SpO2 estimation than horizontal or vertical movements of the hand (M1 or M2). In fact, this is consistent with the clinical findings discussed in [7], which suggested that bending and/or pressing the finger may cause the irregular compression of the vascular bed between the emitter and detector of pulse oximeter sensor, and thus inducing higher errors in the estimated SpO2. A potential solution would be to place multiple sensors around or along the finger so that the ratio of the light intensity received or a pressure reading could be an indication of the degree of bending, pressure exerted or even the level of distortion made on the peripheral blood vascular bed.

Compared with the WMA and FFT approaches, the SPWVD approach showed a significant improvement (p < 0.05) in pulse rate estimation when subjects were in motion. Although such a significant improvement is not found in the estimation of SpO2, this is attributed to the fact that erroneous SpO2 estimates above the 100% upper bound were always rejected. It is hypothesized that when patients with SpO2 much lower than 100% are recruited as subjects for evaluating the different approaches, the performance of each approach will be more notably different from each other. However, this hypothesis remains to be proven in a clinical study involving a significantly large patient population.

## Conclusion

Estimation of SpO2 by a time-frequency representation, the SPWVD, has been investigated in this study. The approach has been tested on four kinds of motions that are found in common movements associated with motion artifacts in pulse oximetry [7,24], i.e. the horizontal movement and vertical movement of the hand, as well as the bending motion and pressing motion of the finger. When compared with the WMA and FFT techniques, the SPWVD approach shows significant improvement (p < 0.05) when subjects bend their finger or press their finger against the sensor. When subjects were in motion, SpO2 levels estimated from the SPWVD, WMA and FFT approaches differed from the reference by -1.07 ± 2.42%, -1.31 ± 3.58% and -1.42 ± 3.18% respectively. The SPWVD approach achieves 0.0% dropout rate and 91% SpO2 PI when subjects were in motion. For the estimation of pulse rate, the SPWVD approach results in a mean absolute pulse rate error of 5.62 bpm, as compared to 16.4 bpm and 11.2 bpm by the WMA and FFT approaches respectively. The results of the study suggested that the SPWVD approach could potentially be used to improve the performance of wearable pulse oximeters by reducing the influence of motion artifacts, in particular when subjects bend their finger or press it against the sensor.

## Competing interests

The author(s) declare that they have no competing interests.

## Authors' contributions

YSY designed and carried out the experiment, analyzed and interpreted the data, and drafted the manuscript. CCYP helped to analyze and interpret the data, and assisted in drafting the manuscript. YTZ conceived of the study, and participated in its design and coordination and helped to finalize the manuscript. All authors read and approved the final manuscript.

## References

[B1] Visram AR, Jones RDM, Irwin MG, Bacon-Shone J (1994). Use of two pulse oximeters to investigate a method of movement artifact rejection using photo-plethysmographic signals. Brit J Anaesth.

[B2] Runciman WB, Webb RK, Barker L, Curriie M (1993). The pulse oximeter: application and limitations: an analysis of 2000 incident reports. Anaesth Intens Care.

[B3] Lawless ST (1994). Crying wolf: false alarms in a pediatric intensive care unit. Crit Care Med.

[B4] Sokwoo Rhee, BH Yang, HH Asada (2001). Artifact-resistant power-efficient design of finger-ring plethysmographic sensors. IEEE Trans Biomed Eng.

[B5] Severinghaus JW, Honda Y (1987). History of blood gas analysis. VII. Pulse oximetry. J Clin Monit.

[B6] Severinghaus JW, Kelleher JF (1992). Recent developments in pulse oximetry. Anesthesiology.

[B7] Tobin RM, Pologe JA, Batchelder PRB (2002). A characterization of motion affecting pulse oximetry in 350 patients. Anesthesia and Analgesia.

[B8] Hayes MJ, Smith PR (2001). A new method for pulse oximetry processing inherent insensitivity to artifact. IEEE Trans Biomed Eng.

[B9] Diab MK (1996). Signal processing apparatus. International Patent Application WO 96/12435.

[B10] Matthews GR (1991). Pulse responsive device. International Patent Application WO 91/18550.

[B11] Parker D (1994). Optical monitor (oximeter, etc) with motion artifact suppression. International Patent Application WO 94/03102.

[B12] Hayes MJ, Smith PR (1998). Artifact reduction in photoplethysmography. Appl Opt.

[B13] Swedlow DB (1994). Oximeter with motion detection for alarm modification. International Patent Application WO 94/22360.

[B14] Scharf JE, Athan S, Cain D (1993). Pulse oximetry through spectral analysis. Proceedings of the Twelfth Southern Biomedical Engineering Conference.

[B15] Rusch TL, Scharf JE, Sankar R (1994). Alternate pulse oximetry algorithms for SpO2 computation. Proceedings of the Sixteenth Annual International Conference of the IEEE Engineering in Medicin and Biology Society.

[B16] Rusch TL, Sankar R, Scarf JE (1996). Signal processing methods for pulse oximetry. Comput Biol Med.

[B17] Dowla FU, Skokowski PG, Leach RR (1996). Neural Networks and Wavelet Analysis in the Computer Interpretation of Pulse Oximetry Data. Proceedings of IEEE Workshop on Neural Networks and Signal Processing.

[B18] Webster JG (1997). Design of pulse oximeters.

[B19] Ricanmato AL, Absher RG, Moffroid MT, Tranowski JP (1992). A time frequency approach to evaluate electromyographic recordings. proceedings of the fifth Annul IEEE symposium on computer based medical systems.

[B20] Velez EF, Absher RG (1989). Smoothed Wigner-Ville parametric modeling for the analysis of nonstationary signals. Proceedings of 1989 International Symposium on Circuits and Systems.

[B21] Hlawatsch F, Boudreaux-Bartels GF (1992). Linear and quadratic time-frequency signal representations. IEEE Signal Processing Magzine.

[B22] Coetzee FM, Elghazzawi Z (2000). Noise-resistant pulse oximetry using a synthetic reference signal. IEEE Trans Biomed Eng.

[B23] Kastle SW, Konecny E (2000). Determine the artifact sensitivity of recent pulse oximeters during laboratory benchmarking. Journal of Clinical monitoring and computing.

[B24] Barker SJ (2002). Motion resistant pulse oximetry: a comparison of new and old models. Anesthesia and Analgesia.

